# Lung transplantation in an intensive care patient with pulmonary alveolar microlithiasis - a case report

**DOI:** 10.12688/f1000research.4035.1

**Published:** 2014-05-28

**Authors:** Bülent Güçyetmez, Aylin Ogan, Aylin Çimet Ayyıldız, Berrin Yalçın Güder, Walter Klepetko

**Affiliations:** 1 Intensive Care Unit, International Hospital, Istanbul, 34149, Turkey; 2 Division of Thoracic Surgery, Medical University of Vienna, Vienna, A-1090, Austria

## Abstract

**Introduction: **Pulmonary alveolar microlithiasis (PAM) is an autosomal recessive disease characterized by the deposition of phosphate and calcium in the alveoli. The disease progresses asymptomatically until later stages. When it becomes symptomatic, lung transplantations performed before the onset of right heart failure may improve life expectancy and quality. Here we present a case report concerning the very first Turkish PAM patient to have undergone lung transplantation surgery.

**Patient information: **A 52 year-old female, Caucasian patient, already diagnosed with PAM in infancy, was admitted to the intensive care unit, diagnosed with pneumonia and hospitalized for 20 days. We decided to refer the patient to a specialized center for lung transplantation. Bilateral lung transplantation was performed in Vienna 14 months later and no recurrence was observed during the first postoperative year.

**Conclusion: **Bilateral lung transplantation may improve both the life expectancy and the quality of life of PAM diagnosed patients with severe respiratory failure who do not suffer from right heart failure. The risk of recurrence should not be considered as a justifying reason to avoid transplantation as a treatment method.

## Introduction

Pulmonary alveolar microlithiasis (PAM) was first described by Harbitz in 1918 ^1^. This rare disease which progresses with calcium and phosphate deposition in the alveolar space is an autosomal recessive disorder caused by the *SLC34A2* gene mutation ^2, 3^. Radiological images reveal typically bilateral, diffuse and symmetrical sandstorm-like widespread radiopaque micronodules ^4^. Turkey is the country with the highest PAM prevalence (16.3%), followed by Italy and USA ^5, 6^. The only known treatment is lung transplantation performed before the onset of right heart failure. No recurrence has been reported after transplantation ^7, 8^. Here we present the case report of the first Turkish patient followed-up in the intensive care unit (ICU) with the diagnosis of PAM, who needed ventilator support at the time of discharge from the ICU and received lung transplantation in Austria.

## Patient information

**Figure 1.**
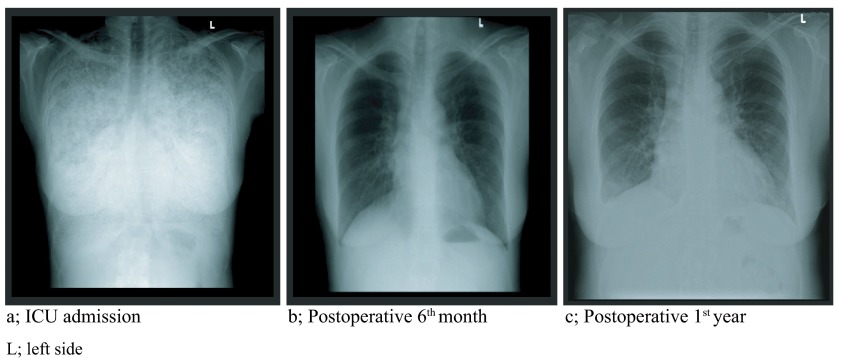
Chest X-rays of the PAM diagnosed patient. **a**; ICU admission (typical chest image of PAM) **b**; 6 ^th^ month after transplantation **c**; 1 ^st^ year after transplantation.

**Figure 2.**
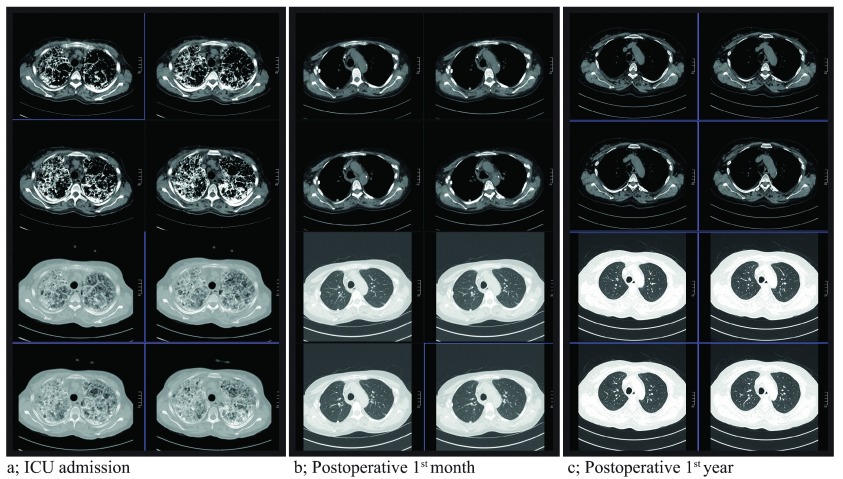
Thoracic CT scans of the PAM diagnosed patient. **a**; ICU admission (bilateral sandstorm image) **b**; 1 ^st^ month after transplantation **c**; 1 ^st^ year after transplantation.

**Figure 3.**
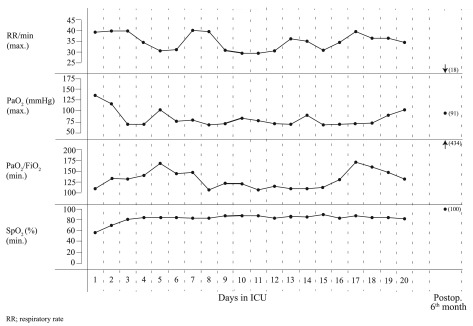
Daily respiratory parameters in ICU.

**Figure 4.**
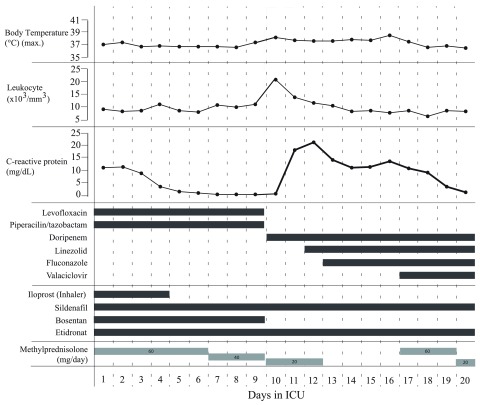
Daily infection markers and therapies in ICU.

A 52 year-old female patient, with a family history of PAM, was diagnosed with the same disease when she was 10 years old and received no treatment or intervention until 2012. The patient affected by PAM presented with tachypnea, exertional dyspnea and fatigue to the Emergency Department and she was admitted to the ICU in 2012 with suspicion of pneumonia. At the time of ICU admission, she was conscious, cooperative and the initial vital signs were SpO _2_: 57% (spontaneous respiration under 5lt/min mask O _2_ support), pulse rate: 127/mn: blood pressure: 126/65mmHg, body temperature: 37°C, C-reactive protein (CRP): 10.83 and leucocyte: 9600/mm ^3^. The arterial blood gas values (10lt/min mask O _2_) were detected as pH:7.45 PaO _2_: 53.5mmHg PaCO _2_: 34mmHg SaO _2_: 86.5% HCO _3_: 24.9 mmol/L base excess: 0.3 mmol/L Na: 133 mmol/L K: 4.8 mmol/L Cl: 106 mmol/L Ca: 1.09 mmol/L lactate: 1.3 mmol/L. The patient was given non-invasive mechanical ventilation (NIMV) support with positive end-expiratory pressure (PEEP): 10cmH _2_O and inspiratory pressure (IP): 22cmH _2_O FiO _2_: 60%. The chest X-ray and thoracic computed tomography (CT) taken at the ICU admission revealed bilateral, diffuse involvement (sandstorm) and decreased aeration areas of both lungs ( Figure 1, Figure 2). The infection markers (body temperature, leucocyte, CRP) of the patient receiving NIMV support during the 20 days of ICU hospitalization improved after the 15 ^th^ day. The daily respiratory parameters (respiratory rate, PaO _2_, PaO _2_:FiO _2_ ratio, SpO _2_), infection markers and the administered medications are shown in Figure 3 and Figure 4. Since the patient still had the consistent need of NIMV support despite the improvement of the laboratory values, and no changes were detected in the radiological images, she was evaluated together with Pulmonary Diseases and Cardiology departments. Following thoracic CT, echocardiography (mild pulmonary hypertension, EF 60%) and respiratory function tests (Forced Expiratory Volume in 1 second, FEV _1_:0.51L Forced Vital Capacity, FVC:0.54L FEV _1_/FVC:0.94), the patient was discharged from the ICU on the 20 ^th^ day and referred to a specialized lung transplantation center. No extrapulmonary involvement was observed by Positron Emission Tomography (PET). The patient underwent bilateral lung transplantation in Vienna 14 months after initial admission (she was under oxygen and NIMV support during these 14 months). She was followed-up during the first seven postoperative days in the ICU and discharged on the 21 ^st^ day from the hospital. On the postoperative 6 ^th^ month, the values of the patient, having no need for oxygen or NIMV support, were FEV _1_:2.21 FVC:2.26 FEV _1_/FVC:98%. The results of chest X-rays taken on the postoperative 1 ^st^ month and 1 ^st^ year, the thoracic CT scans taken on the postoperative 6 ^th^ month and 1 ^st^ year and the arterial blood gas under room air on the postoperative 6 ^th^ month of the patient administered mycophenolic acid 760mg/day, tacrolimus 0.5mg/day, and prednisolone 5mg/day medication are demonstrated in Figure 1, Figure 2 and Figure 3 respectively.

## Discussion

The etiology, epidemiology, clinical findings and typical radiological images related to PAM disease have been almost completely documented. The aim here is to discuss the advantages of lung transplantation as a treatment option for PAM.

It has been described that PAM is an autosomal recessively inherited disorder related to genetic factors ^2, 3^. Although PAM is rarely observed in infants ^9^, the clinical findings and the radiological changes advance progressively over-time; micronodular structure (sandstorm) develops due to the deposition of calcium and phosphate, aeration areas decrease, fibrosis increases and hypoxemia occurs. Patients presenting to the hospital with these clinical findings are generally over the age of 40 and no administered treatments result in full recovery. Systemic corticosteroids, calcium-chelating agents and bronchoalveoler lavage (BAL) are palliative solutions ^10^. Ozçelik *et al.* have described the positive effects of the long term use of sodium etidronate which is effective by inhibiting the hydroxyapatite microcrystal formation in pediatric patients ^11^. However, there are also some studies showing that the sodium etidronate treatment is ineffective ^7^.

The priority for these patients admitted to the ICU should be to seek solutions for recovering hypoxemia. It is observed that patients presenting highly decreased aeration areas have already undergone many treatment methods ^11^. In Figure 4 we show that the patient was administered sodium etidronate, methylprednisolone and sildenafil in the ICU. Besides, no improvement was observed in the radiological images or respiratory parameters despite the oxygen and NIMV support and the regress in the infection markers. Even at the time of discharge from the ICU, the patient was under NIMV support, and was oxygen dependent with a peripheral saturation of 90%, PaO _2_ 90–95mmHg and with PaO _2_/FiO _2_ ratio under 150. The patient clinically worsened with infection; after the regression of the infection, she could only return to her previous hypoxemic condition despite NIMV support.

The issue that intensivists should discuss is whether a patient with a hypoxemic condition should be discharged with the recommendation of oxygen and NIMV support or whether the transplant choice should be brought forward.

Although Turkey is the country with the highest PAM prevalence, we have not encountered any published reports on transplanted cases upon the diagnosis of PAM in Turkey. Lung transplantation is a treatment option for PAM patients and is recommended in severe cases of oxygen dependent respiratory failure before the onset of right heart failure ^7, 8, 10^. Bonette *et al.* recommended bilateral lung transplantations instead of unilateral transplants on the grounds of resistant shunt development in a lung transplantation series of 14 cases, one of which was caused by PAM ^12^. Besides, no recurrence was detected in the presented cases or series ^7, 8, 10, 13, 14^. Moon *et al.* reported the case of a patient who survived for 15 postoperative years with no recurrence after the lung transplantation was performed after PAM diagnosis ^8^. In their series, Shigemura *et al.* reported no recurrence and only two cases of postoperative major bleeding after bilateral lung transplantation ^15^. Furthermore, they reported a significant increase in the FEV _1_ and FVC levels of the patients and considered bilateral lung transplantation in PAM cases as a successful and reliable treatment method.

Thus, considering lung transplantation as a treatment method in PAM cases instead of medication or artificial respiratory support treatments, both of which are known to be ineffective, seems rational. In our case, we discussed the lung transplantation indication of this PAM-diagnosed patient to offer her a chance of full recovery. The differences observed between the preoperative and postoperative periods demonstrated the importance of performing bilateral lung transplantation in such cases regardless of the recurrence risk. Not only did the radiological follow-up for one year show no recurrence; but also the patient receiving immunosuppressive treatment no longer needed oxygen or artificial respiratory support systems.

## Conclusion

Bilateral lung transplantation may improve both the life expectancy and quality of PAM-diagnosed patients with severe respiratory failure who do not suffer from right heart failure. The risk of recurrence should not be considered as a valid reason to eliminate transplantation option as a treatment method.

## Key messages

- All the other treatment methods in PAM are palliative except for transplantation.
- A PAM-diagnosed patient being followed-up in the ICU due to severe respiratory failure needs oxygen and NIMV support even at the time of discharge.
- Intensivists should discuss the transplantation option in cooperation with the pulmonologists, cardiologists and transplantation team; they should also have an active role in the management of the PAM-diagnosed patients after their discharge.
- The risk of recurrence should not be considered as a justifying reason to avoid the transplantation option.

## Consent

Written informed consent for publication of this case report and accompanying figures was obtained from the patient.
