# Supplementary material for: Lung transplantation in an intensive care patient with pulmonary alveolar microlithiasis - a case report
Source: F1000Res. 2014 May 28;3:118. [Version 1] doi: 10.12688/f1000research.4035.1 (PMC4133765; doi:10.12688/f1000research.4035.1)
Supplement: Peer review history

# Peer review: Referee response for version 1

**DOI:** 10.5256/f1000research.4322.r5518

**Published:** 2014-07-24

**Authors:** Johan Groeneveld

**Affiliations:** 1 Department of Intensive Care, Erasmus Medical Center, Rotterdam, Netherlands

Well documented case report. I wonder whether patients with mild cor pulmonale would also qualify for this life saving procedure, since this may ameliorate further remodeling of the heart. Another thing that bothers me is whether this patient was on selective decontamination of the digestive tract during postoperative ICU stay or not? The documentation of pulmonary infections and their (appropriate) antibiotic treatment is somewhat lacking. This is an important issue for lung transplant, immune suppressed patients. Did this patient harbor multi-resistant microorganisms ****prior to transplant?

I have read this submission. I believe that I have an appropriate level of expertise to confirm that it is of an acceptable scientific standard.

# Peer review: Referee response for version 1

**DOI:** 10.5256/f1000research.4322.r5369

**Published:** 2014-07-07

**Authors:** Michael O'Connor

**Affiliations:** 1 Department of Anesthesia and Critical Care, University of Chicago, Chicago, IL, USA

This case report represents the state of the art in the care of these patients very well. This patient was admitted to the hospital with life threatening respiratory failure. The underlying cause was diagnosed and treated. An excellent discharge plan was created and implemented. The patient was referred for lung transplantation, and underwent the procedure with an apparently good functional result. The duration of follow up is relatively short.

I have read this submission. I believe that I have an appropriate level of expertise to confirm that it is of an acceptable scientific standard.
